# UV-B Induces Distinct Transcriptional Re-programing in UVR8-Signal Transduction, Flavonoid, and Terpenoids Pathways in *Camellia sinensis*

**DOI:** 10.3389/fpls.2020.00234

**Published:** 2020-03-03

**Authors:** Lubobi Ferdinand Shamala, Han-Chen Zhou, Zhuo-Xiao Han, Shu Wei

**Affiliations:** ^1^State Key Laboratory of Tea Plant Biology and Utilization, Anhui Agricultural University, Hefei, China; ^2^School of Life Sciences, Anhui Agricultural University, Hefei, China; ^3^Tea Research Institute, Anhui Academy of Agricultural Sciences, Huangshan, China

**Keywords:** ultraviolet radiation, UVR8 photoreceptor, abiotic stress, phenolics, HY5, terpenoids metabolism, climate change, flavonoid accumulation

## Abstract

Plants are known to respond to Ultraviolet-B radiation (UV-B: 280–320 nm) by generating phenolic metabolites which absorbs UV-B light. Phenolics are extraordinarily abundant in *Camellia sinensis* leaves and are considered, together with pleasant volatile terpenoids, as primary flavor determinants in tea beverages. In this study, we focused on the effects of UV-B exposure (at 35 μW cm^–2^ for 0, 0.5, 2, and 8 h) on tea transcriptional and metabolic alterations, specifically related to tea flavor metabolite production. Out of 34,737 unigenes, a total of 18,081 differentially expressed genes (DEGs) due to UV-B treatments were identified. Additionally, the phenylpropanoid pathway was found as one of the most significantly UV-B affected top 20 KEGG pathways while flavonoid and monoterpenoid pathway-related genes were enhanced at 0.5 h. In the UVR8-signal transduction pathway, *UVR8* was suppressed at both short and long exposure of UV-B with genes downstream differentially expressed. Divergent expression of *MYB4* at different treatments could have differentially altered structural and regulatory genes upstream of flavonoid biosynthesis pathways. Suppression of *MYB4-1&3* at 0.5 h could have led to the up-regulation of structural *CCOAOMT-1&2, HST-1&2, DFR-4, ANR-2*, and *LAR-1&3* genes resulting in accumulation of specialized metabolites at a shorter duration of UV-B exposure. Specialized metabolite profiling revealed the correlated alterations in the abundances of catechins and some volatile terpenoids in all the treatments with significant accumulation of specialized metabolites at 0.5 h treatment. A significant increase in specialized metabolites at 0.5 h treatment and no significant alteration observed at longer UVB treatment suggested that shorter exposure to UV-B led to different display in gene expression and accumulation of specialized metabolites in tea shoots in response to UV-B stress. Taken together, our results indicated that the UV-B treatment applied in this study differentially altered the UVR8-signal transduction, flavonoid and terpenoid pathways at transcriptional and metabolic levels in tea plants. Our results show strong potential for UV-B application in flavor improvement in tea at the industrial level.

## Introduction

Tea is a widely consumed non-alcoholic beverage, made from tender tea leaves (*Camellia sinensis*) (Banerjee, 1992), which contain substantial volatiles and extraordinarily high amount of polyphenols (16–30%, dry weight) (Graham, 1992) such as epigallocatechin-3-gallate (EGCG) and epicatechin-3-gallate (ECG). These specialized compounds are crucial for tea brisk, umami taste, pleasant scent, and anticancer, antibacterial, and immunostimulant effects (Kumazawa, 2006; Yu et al., 2014; Han et al., 2016).

Metabolic characteristics of tea plants might be evolutionally developed and associated with the environments through the course of evolution. *C. sinensis* is native to southwest Asia and is grown at high altitudes (Meegahakumbura et al., 2018), receiving high fluence rates of UV-B radiation (280–320 nm) compared to plant species grown in low altitude regions (Madronich et al., 1995; Casati et al., 2006). The impact of UV-B on plant growth and development is largely dependent on irradiation dose and plant species (Casati and Walbot, 2003; Jenkins, 2009; Pontin et al., 2010). UV-B can regulate gene expression both by UV-B specific and non-specific pathways(Jenkins, 2009). High fluence of UV-B may activate both UV-B specific and non-specific pathways leading to the generation of reactive oxygen species (ROS), DNA and protein damage, inhibition of photosynthetic reactions and protein synthesis (Jansen et al., 1998; Frohnmeyer and Staiger, 2003), and consequently leading to a wide array of changes at molecular and morphological levels in plants (Mackerness et al., 1999; Brown et al., 2005; Meegahakumbura et al., 2018). Ambient UV-B at low fluence rates has regulatory effects on plant growth and development, biochemical composition (Boccalandro, 2001; Suesslin and Frohnmeyer, 2003) and affect the expression of a wide range of genes (Casati and Walbot, 2003; Casati and Walbot, 2004; Jenkins, 2009). These UV-B specific alterations in plants are mediated by the signaling transduction module of UV RESISTANCE LOCUS8 (UVR8) and COP1 (Rizzini et al., 2011; Jenkins, 2017), which contains multiple transcription factors such as ELONGATED HYPOCOTYL5 (HY5) (Binkert et al., 2014), and MYB family members (Stracke et al., 2009). UV-B induced metabolomic changes (including enhanced flavonoids, anthocyanins, and many other phenolics) have been reported in some plant species (Takshak and Agrawal, 2016). Correspondingly, UV-B enhanced transcriptional alteration has also been reported in multiple structural and regulatory genes in phenylpropanoid/flavonoid pathways such as *PHENYLALANINE AMMONIA-LYASE* (*PAl*), *CHALCONE SYNTHASE* (*CHS*), *DIHYDROFLAVONOL 4-REDUCTASE* (*DFR*), *FLAVANONE-3-HYDROXYLASE* (*F3H*), *MYB12* and *MYB111* (Brown et al., 2005; Stracke et al., 2009) and other transcription factors of the regulatory MYB/bHLH/WD40 (MBW) complexes, which regulate phenylpropanoid biosynthesis (Xu et al., 2015; Li et al., 2017). Interestingly, phenolic metabolites including diverse flavonoids, which are deposited in plant epidermal tissues (Greenberg et al., 1996; Rozema et al., 1997), absorbing light in the wavelength ranges 250–270 nm and 330–350 nm (Kumar et al., 2018) with an absorption peak around 300 nm (Gábor, 2013), function as a UV-absorbing sunscreen and, in turn, offer protection to plants from UV-B damage (Li et al., 1993; Brown et al., 2005; Liu et al., 2015). A recent study applying UV-B treatment to tea plants revealed their distinct responses compared to some other plants (Liu L. et al., 2018). For example, only EGCG was induced, while the abundance of other catechins remains unchanged. In addition, the genes (*UVR-8*, *COP1*, *HY5*) in the UV-B signaling transduction module are not consistently UV-B up-regulated (Liu L. et al., 2018) as found in many other plant species (Rizzini et al., 2011; Jenkins, 2017).

Pleasant and characteristic aroma of teas is highly demanded and considered as a key indicator for good quality of teas. Tea aroma compounds are commonly generated from various metabolite precursors during the manufacturing process (Ho et al., 2015). In tea leaves biosynthesis of volatile terpenoids, which impart teas with floral scent, is an essential prerequisite for the accumulation of volatile terpenoid conjugates and volatile release after conjugate hydrolysis. In plants, diverse terpenoid compounds are known synthesized through the condensation of the terpenoid building blocks dimethylallyl pyrophosphate (DMAPP) and isopentenyl pyrophosphate (IPP) generated from the mevalonate (MVA) and the 2-*C*-methyl-D-erythritol 4-phosphate (MEP) pathways, respectively (Hemmerlin et al., 2012). Biosynthesis of different terpenoids is catalyzed using prenyl diphosphate precursors by different terpene synthases *(TPS)* (Chen et al., 2011). Recently a bifunctional gene generating two transcripts coding a linalool synthase and a nerolidol synthase, respectively was reported in tea and the transcript for linalool synthase alone was induced by methyl jasmonic acid (Liu G.F. et al., 2018). Additionally, a few *TPS* genes such as nerolidol synthase genes were also characterized in tea plants (Zhou et al., 2017; Liu G.F. et al., 2018). Moreover, supplemental UV-B radiation to harvested tea shoots for 2 h in the manufacturing process can enhance the abundance of many odorants including linalool in the resulting tea (Jang et al., 2010). In addition, some *TPS* genes were found being activated by UV-B irradiation in peach (Liu et al., 2017). Therefore, it would be interesting to find out whether different time duration (0, 0.5, 2, and 8 h) of UV-B irradiation, rather than stress conditions of the excised leaves, can affect the expression of *TPS* genes in the intact tea leaves.

Specialized metabolites synthesis in plants is affected by many endogenous and exogenous factors; however, transcriptional regulation plays a key role and thus becomes a subject for better understanding the molecular mechanisms underlying metabolic flux regulation (Patra et al., 2013). It is well established that tea leaves are rich in UV-B absorbing polyphenolics, such as catechins (Graham, 1992). Hence, it would be interesting to investigate whether UV-B induced transcriptional responses of tea plants are distinct from those of other plant species with low polyphenolic abundance, with an emphasis on those genes related to the flavonoid pathways for a better understanding of the UV-B transcriptional regulation of polyphenolic production.

This study was designed to clarify UV-B induced alterations in gene transcription and metabolites related to tea flavor determinants (catechins and volatiles) (Zheng et al., 2008; Jang et al., 2010) for theoretical understanding of transcriptional regulation of flavor determinant formation and practical improvement of green tea beverage flavor. Thus UV-B radiation was supplemented to tea plants with different exposure durations and transcriptional and metabolic profiling was performed with an emphasis on UVR8-signal transduction, flavonoid, and volatile terpenoid pathways to determine a potential transcriptional control of tea flavor-determinant production. Our findings open a window for future studies on the physiological and ecological functions of volatiles terpenoids in response to environmental-related stress in the era of climate change.

## Materials and Methods

### Plant Materials and UV Treatments

One-year-old potted plants of *Camellia sinensis* cv. “Longjing-43” were planted in the tea farm of Anhui Agricultural University located at 31°55′ 42.8″ N, 117°12′ 09.1″ E, Hefei, China. For UV-B treatments, an effective dose rate at ≅35 μW cm^–2^ irradiances at 311 nm normalized UV-B was provided according to Jang et al. (2010) for four different exposure durations (0, 0.5, 2, and 8 h). For each exposure treatment with biological triplicates, 10 plants were applied for each replicate. The UV-B radiation was generated using a TL 100W/01 tube (311 and 313 nm spectrum peaks, Philips, Germany) in laboratory condition under the constant white light (100 μmol⋅m^–2^⋅s^–1^) delivered by LEDs (Tops 10 Power Pure White Led OSW4XAHAE1E) 2.7 m) above the plants. The UV-B tube was placed 40 cm above the plants and the irradiation area was 0.5^∗^1.8 m as described earlier (Jang et al., 2010). The group set as a control (0 h) was placed under white light and exposed to the same UV-B tube but covered with a 120 μm clear polyester plastic filter (Hangzhou Phillis Filter Tech Co., Ltd., Hangzhou, China), which absorbed more than 95% of UV-B without affecting photosynthetically active radiation (PAR). After UV-B exposure, two or three shoot tips with two folded leaves were collected from each treated or control plant and immediately frozen in liquid nitrogen and maintained at –80°C for RNA extraction and metabolite analysis. Totally, 12 samples were obtained.

### RNA-Seq Library Construction and Illumina Sequencing

Plant samples subjected to different durations of UV-B exposure (0, 0.5, 2, and 8 h) were used to extract total RNA using RNAprep pure Plant Kit (TianGen Biotech., Ltd., Beijing, China). The RNA integrity was examined using both agarose gel electrophoresis and a Nanodrop 2000 spectrophotometer (Thermo Fisher Scientific, Wilmington, DE, United States). For RNA-seq analysis, equal amounts of RNA extract was obtained from all the 12 samples pooled together and sent to Shanghai OE Biotech (Shanghai, China) for cDNA library construction and sequencing to generate transcriptomic data for this study. Enrichment of mRNA, fragmentation, the addition of adapters, size selection, PCR amplification, and RNA-Seq was performed by staff at Shanghai OE Biotech (Shanghai, China). mRNA was first enriched from 20 μg total RNA using magnetic beads with Oligo (dT) (Invitrogen, Beijing, China) and then fragmented, followed by cDNA synthesis using these short fragments as templates and random primers (Takara). Then, the double-stranded cDNA fragments were ligated to adapters using T4 DNA ligase (Invitrogen, United States). The two final cDNA libraries were constructed after PCR enrichment of these ligated products. Multiplexed RNA-Seq libraries were sequenced using the Illumina HiSeqTM 2000 platform (Illumina, Inc., Shanghai, China). Moreover, *De novo* assembly of RNA-Seq reads and functional annotation of the unigenes were also conducted using the transcriptome assembler Trinity (Grabherr et al., 2011). Unigene sequences obtained from the UV-B treated and non-treated control were aligned using BLASTX against the databases of GO (Gene Ontology) and KEGG (Kyoto Encyclopedia of Genes and Genomes). All sequencing data were deposited in the National Center for Biotechnology Information (NCBI) Sequence Read Archive (accession number PRJNA597433).

### RNA-Seq Data Analysis

For the identification of high-quality sequencing data and clean reads, quality control was performed on the raw reads. Filtered clean reads from each of the 12 samples were independently aligned to the reference transcripts of tea genome (Wei et al., 2018) with the help of Bowtie2 software (Langmead et al., 2009) and used to approximate the abundance of gene transcripts using the RSEM method (Li and Dewey, 2014) measured as fragments per kilobase of transcript per million fragments sequenced (FPKM) (Trapnell et al., 2010). Based on method described by Audic and Claverie (1997), differentially expressed genes (DEGs) were identified. False discovery rate (FDR) of >0.001 and an absolute value of FPKM fold-change of >2 as the thresholds were used to examine the significance DEGs. Wilcoxon signed-rank test was used to analyze the difference in gene expression among the groups and to evaluate the influence of UV-B on the transcriptional level of genes in tea shoots. *P*-value < 0.05 was considered statistically significant (Supplementary Figure S3), and a heat map of expression values was generated with the help of the T-MeV 4.9.0 software (Howe et al., 2011). Annotation analyses of DEGs were performed using WEGO software (Ye et al., 2018) for GO term functional classification, and pathway enrichment analysis of DEGs was performed based on the Kyoto Encyclopedia of Genes and Genomes (KEGG) database.

### HPLC Analysis for Catechins and Caffeine

Tea samples used in this study were freeze-dried for catechin and caffeine analysis. The sample (0.5 g) was placed into a 100 mL vial with 20 mL boiled water in a 90°C water bath for 5 min, and then was filtered and quantified according to Han et al. (2016) with some modifications. In brief, an HPLC (600 Controller, 2489 UV/Visible Detector; Waters, Milford, MA, United States) equipped with a C18 column (5 μm × 4.6 mm × 250 mm; Phenomenex, Torrance, CA, United States) was used. The compounds were quantified (mg/g DW) using calibration curves determined from authentic standards as described before (Feng et al., 2014). Data were statistically analyzed using one-way analysis of variance (*t*-tests, *p* < 0.05). Chemical analyses had three biological replicates.

### GC-MS Analysis for Terpenoid Volatiles

Gas chromatography (Agilent 7697A)/mass spectrometry (Agilent 7890A) (GC/MS) and a DB-5 capillary column (30 m × 0.25 mm × 0.25 μm; Agilent) were used in this study. Chemicals were identified by comparing the retention time and mass spectrum either with those of authentic standards or NIST mass spectral library version 17. Compounds were quantified based on the calibration curves established using a series of diluted solutions prepared with authentic standards (Han et al., 2016). The concentrations of the volatiles were expressed as relative content in percentage. Data were also statistically analyzed using one-way analysis of variance (*t*-tests, *p* < 0.05). The repeatability of the analytical method was evaluated using triplicates of independently prepared samples as for catechin analysis.

### Quantitative Real-Time PCR Assay

For studying the UV-induced transcriptional alterations in tea plants, the qPCR analysis was performed on a CFX96 platform (Bio-Rad, www.bio-rad.com/) and the Top Green qPCR SuperMix (TransGen Biotech, Beijing, China) according to the manufacturer’s instructions. Expression of annotated genes in UVR8-signal transduction, phenylpropanoids, volatile terpenoids pathway and *TPS* genes in the UV-B treated tea shoots for 0, 0.5, 2 and 8 h treatment were quantified using gene-specific primers (Supplementary Table S2). QPCR program was computerized as follows: 95°C for 10 min; 40 cycles of 95°C for 10 s, annealing at 58°C for 15 s, and elongation at 72°C for 34 s; followed by a heat dissociation protocol from 55°C to 95°C. PCR reaction efficiencies for all test genes were over 90% and their transcript levels were normalized using the reference gene 18s RNA, then calculated according to the 2^–ΔΔ^ ^*Ct*^ method (Livak and Schmittgen, 2001). Dissociation curves for each amplicon were evaluated to verify the specificity of each amplification reaction. All the qPCR analyses were carried out using three biological replicates each containing technical triplicate and the data were subjected to analysis using SPSS 17.0 statistical package.

## Results

### RNA-Seq Analysis Reveals Re-programing of Global Transcription in the Tea Shoots in Response to UVB Exposure

**FIGURE 1:**
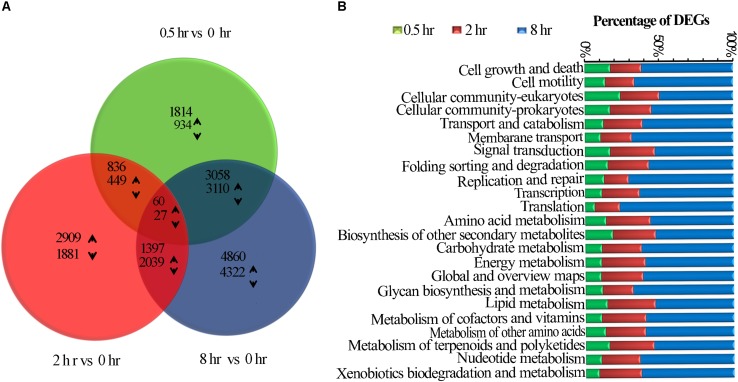
UVB induced transcriptomic changes in tea shoots. **(A)** Venn diagram showing the statistics of the number of common and unique differentially expressed genes between different comparison groups (0.5 h Vs. 0 h, 2 h Vs. 0 h, and 8 h Vs. 0 h (control) treatments. The numbers of differentially expressed transcripts are provided as down-regulated and up-regulated Using arrows. **(B)** The number of all the DEGs in the entire KEGG pathway affected in the three treatments.

RNA-seq analysis from all tea samples resulted in 91.03G clean data with 6.89∼8.07G for each sample and the Q30 base distribution ranged from 95.06 to 95.94%. The rate of the alignment between sequencing reads and the reference genome (Wei et al., 2018) fell in a range of 92.14∼94.17%. Further, a total of 23,888 unigenes and 12,307 differentially expressed genes (DEGs) were identified based on method described by Audic and Claverie (1997), differentially expressed genes (DEGs) were identified (Supplementary Figure S3). False discovery rate (FDR) of >0.001 and an absolute value of FPKM fold-change of >2 as the thresholds were used to examine the significance DEGs (Table 1 and Supplementary Table S1). All DEGs were visualized in a Venn diagram for differential transcriptional alterations due to different UV-B exposure durations. The highest number of DEGs was observed at 8 h treatment (8 h vs. 0h) while the lowest at 0.5 h (0.5 h vs. 0 h) treatment. Sixty up-regulated and twenty-seven down-regluated DEGs were found common to all the UV-B treated groups (Figure 1A). Numerous pathways of KEGG associated with DEGs were UV-B affected. Among the affected KEGG pathways categorized into cellular processes, environmental information processes, genetic information processes and metabolism, the majority of DEGs were associated with metabolism processes. Biosynthesis of other specialized metabolites was mostly affected after glycan biosynthesis and metabolism at 0.5 h as compared to other pathways at the same exposure time. Metabolism of terpenoids and polyketides were affected by all the treatments with significant effects at 8 h treatment (Figure 1B).

**TABLE 1:** Summary of RNA-Seq statistical assembly.

**Assembly**				

**Total raw reads**	**Total clean reads**	**Total clean bases (G)**	**Q30%**	**GC%**
662,300,000	644,490,000	97.5064	95.55%	45.02%

**Annotation**				

	**GO**	**DEGS**	**KEGG**	**DEGS**

Treatments	Unigenes		Unigenes	
0.5 hr	23888	1929	10849	755
2 hr		3495		1570
8 hr		6883		3449

**FIGURE 2:**
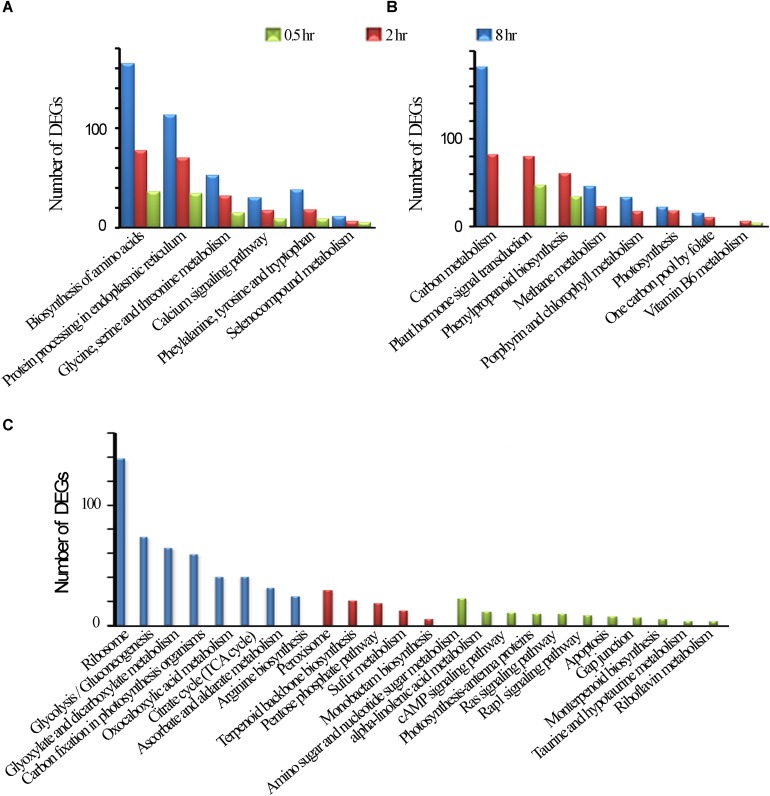
KEGG Enrichment top 20. **(A)** common DEGs in all the three treatments. **(B)** Common DEGs to the two treatments. **(C)** Unique DEGs to each of the three treatments.

Among UV-B induced top 20 affected KGG pathways, six pathways were affected by all the three treatments, including amino acid biosynthesis and protein processing in endoplasmic reticulum (Figure 2A). Phenylpropanoid biosynthesis was noted in addition to plant hormone signal transduction, carbon metabolism, photosynthesis which were among the eight pathways affected by the two treatments only (Figure 2B). Monoterpenoid biosynthesis together with Rap1 and Ras signaling pathways was noted among the 11 affected pathways unique to 0.5 h treatment. In addition, upregulated examples for UV-B irradiated shoots include 8 and 4 genes involved in flavonoid and monoterpenoid pathways, respectively at 0.5 h (Supplementary Table S5). Ribosome was the most affected pathway unique to 8 h treatment among other 7 pathways including citrate cycle. We observed the least number of pathways unique to 2 h treatment with perixisome and terpenoid backbone biosynthesis as the most affected (Figure 2C). As expected, some stress-related pathways were also UV-B affected such as response to oxidative stress, UV-B signaling transduction, and stimuli to light, bacteria, fungi, and multiple hormones (Supplementary Figure S1 and Supplementary Table S4).

It is interesting to note that some pathways such as “Glycine, serine and threonine metabolism,” “Phenylalanine, tyrosine and tryptophan,” “Biosynthesis of amino acids,” Ribosomes and “Protein processing in endoplasmic reticulum” were significantly affected when a longer exposure duration (for 8 h) was applied, while other pathways such as “Plant hormone signal transduction, Phenylpropanoid biosynthesis” were affected when exposed for 2 h, and 0.5 h of UV-B exposure whiles others such as “Photosynthesis, Methane metabolism,” and “Plant hormone signal transduction”, were more pronouncedly affected for a longer exposure period (Figures 2A–C and Supplementary Table S3).

### Differentially Expressed Genes in UVR8-Mediated Signal Transduction Pathway

**FIGURE 3:**
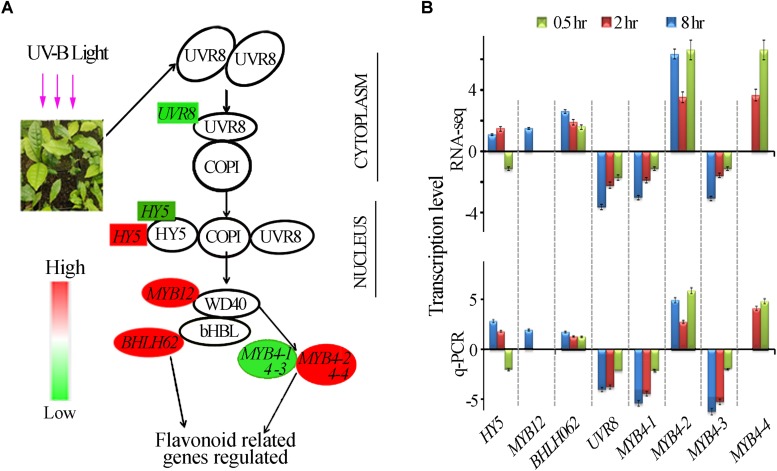
UVR8- mediated signal transduction pathway. **(A)** Differential expression of the genes in the UVR8-signal transduction pathway revealed by transcriptomic data. Genes highlighted in red and green were significantly enhanced and suppressed respectively. **(B)** Gene expression in UVR8 signaling pathway analyzed by RNA-seq and qPCR.

*UVR8* is a UV-B receptor and mediates UV-B induced plant responses through signal transduction pathway (Kliebenstein et al., 2002). To understand roles of *UVR8* and its signal transduction pathway in tea plants in response to UVB exposure at different time periods, transcriptional alteration of the genes in this pathway were examined in this study. We identified only one UVR8 transcript in our transcriptomic data even though 10 more UVR8 transcripts were retrieved from tea genome after performing a local blast. Our data showed that in all the UV-B treated tea plants, *UVR8* transcription levels were down-regulated (Figure 3). Transcription levels of *HY5*, *MYB4*, *bHLH62*, and *MYB12*, transcriptional factors regulating the downstream structural genes, were all affected differently at different exposure times (Figure 3A). Here, we observed *HY5* was down-regulated at 0.5 h and up-regulated at 2 and 8 h treatments. Four *MYB4* (*MYB4-1, MYB4-2, MYB4-3*, and *MYB4-4*) were observed to be altered differently through all the treatments while *MYB12* and *bHLH* 62 were up-regulated at 8 h treatment (Figure 3B). Transcript abundance of 8 selected genes in this pathway was validated using real-time qPCR analysis. Consistent UV-B responses at different exposure time were observed between the RT-qPCR analysis and RNA-Seq gene expression (Figure 3B). UVR8 transduction pathways responded to UVB exposure leading to suppression of *UVR8* gene and differential expression of genes downstream UV8. Further work need to be done to find out the role of other 10 *UVR8* genes in tea plant.

### UV-B Induced Differential Expression of Genes in the Flavonoid Biosynthesis Pathway

**FIGURE 4:**
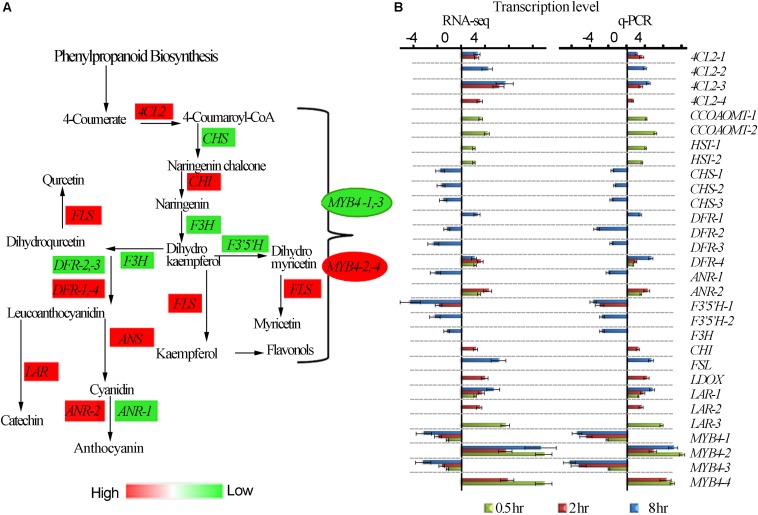
UV-B induced differential expression of flavonoid genes. **(A)** Differential expression of flavonoid pathway genes revealed by transcriptomic data. **(B)** Shows RNA-seq and q-PCR gene expression in pheneylpropanoid pathway. *4CL2*, *4-COUMARATE:COA LIGASE*; *CHS*, *CHALCONE SYNTHASE*; *F3H*, *FLAVANONE 3-HYDROXYLASE*; *F3′H*, *FLAVANOID 3′-HYDROXYLASE*; *H3′5′H*, *FLAVANOID 3′,5-HYDROXYLASE*; *DFR*, *DIHYDROFLAVONOL 4-REDUCTASE*; *FLS*, *FLAVONOL SYNTHASE*; *ANR*, *ANTHOCYANIDIN REDUCTASE*; *ANS*, *ANTHOCYANIDIN SYNTHASE; F5H*, *FERULATE 5-HYDROXYLASE*. *CCOAOMT – CAFFEOYL-COA O-METHYLTRANSFERASE; HST-HYDROXYCINNAMATECOA SHIKIMATE TRANSFERASES; LDOX – LEUCOANTHOCYANIDIN DIOXYGENASE.*

It has been widely reported that UV-B irradiation induces flavonoids and anthocyanins accumulation which acts as UV-B protectant in different plant species. The tea plant is classified as extraordinary plant species due to its enormous accumulation of flavonoids (Punyasiri et al., 2004). We examined the effect of different time exposure of UV-B on tea shoots using transcriptomic data (Figure 4). Thirty transcripts were differentially affected in this pathway with 11 down-regulated and 19 up-regulated. We observed up-regulation of transcripts *4CL2-1, 4CL2-3* were up-regulated at 2 and 8 h treatment while *4CL2-2* and *4CL2-4* were upregulated at 8 and 2 h treatment, respectively. *DFR-4, ANR-2, CHI, FSLI, ANS, LAR-1, LAR-2*, and *LAR-3* at 2 and 8 h exposure periods. *CCOAOMT-1, CCOAOMT-2, HST-1, HST-2, ANR-2, DFR-4, LAR-1* and *LAR-3* were up-regulated at 0.5 h exposure consistent with observation made earlier in flavonoid synthesis pathway. *CHS-1, CHS-2, CHS-3, DFR-1, DFR-2-DFR-3, ANR-1, F3′5′H-1, F3′5 ′H-2*, and *F3′H* were all down-regulated at 8 h exposure time. Moreover, divergent expression patterns of some genes in phenylpropanoid pathway including transcriptional factor *MYB4* were also noted (Figure 4A and Supplementary Table S5). *MYB4-1* and *MYB4-3* were all down-regulated by all the three treatments while *MYB4-2* was up-regulated in all the treatments. It was interesting to note suppression of most structural genes at 8 h treatment but no changes were observed in 0.5 and 2 h treatment. Up regulation of *MYB4-2* at 8 h treatment could have suppressed the structural genes as observed at 8 h treatment. *MYB4-4* was up-regulated at 0.5 and 2 h treatment, but such an enhancement did not exert any detectable regulatory effects on its target genes except for *F3′5′H-1*, in flavonoid pathway. Down regulation of *MYB4-1* and *MYB4-3* at 8 h treatment could have led to upregulation of structural genes *4CL2-1, 4CL2-2, 4CL2-3, 4CL2-4, DFR-4, ANR-2, CHI, FSLI, ANS, LAR-1, LAR-2*, and *LAR-3* (Figure 4B). Analysis of MYB4 protein sequences revealed differences in their structures (Supplementary Figure S2). The up-regulation and down-regulation of *MYB4* could be the reason for divergent expression of genes in flavonoid pathway.

To validate the reliability of gene expression analysis in this study, 26 genes with potential roles in flavonoid biosynthesis, were selected for real-time qPCR analysis. Our real-time qPCR data showed that these genes expression patterns, matched the gene expression patterns observed in RNA-Seq analysis (Figure 4B).

### UV-B Induced Differential Expression of Genes in Terpenoid Biosynthesis Pathway

**FIGURE 5:**
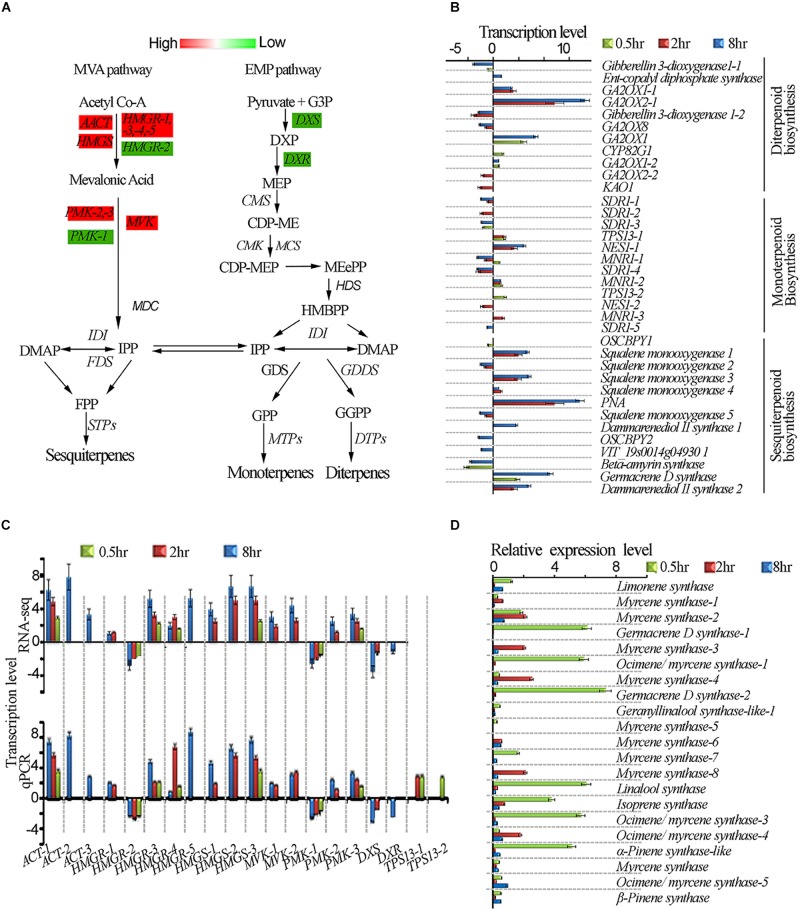
UV-B induced enhancement of terpenoid biosynthesis pathway. **(A)** Differential expression of terpenoid pathway genes revealed by transcriptomic data. **(B)** Shows genes affected in diterpenoid, monoterpenoid and sesquiterpenoid biosynthesis pathways. **(C)** Shows RNA-seq and qPCR gene expression in terpenoid pathway. **(D)** qPCR gene expressions of selected *TPS* genes in terpenoid pathway. *Cs18srRNA* was employed as a reference gene for data normalization using 2^–ΔΔCt^ method. *HMGS*, *3-HYDROXY-3- GLUTARYL COENZYME A SYNTHASE*; *HMGR*, *3-HYDROXY-3-METHYL-GLUTARYL COENZYME A REDUCTASE*; *DXS*, *1-DEOXY-D-XYLULOSE-5-PHOSPHATE SYNTHASE*; *CMK*, *4-CYTIDINE 50-DIPHOSPHO-2-C-METHYL-D-ERYTHRITOL KINASE*; *HDS*, *HYDROXY-2-METHYL-2-(E)-BUTENYL 4-DIPHOSPHATE SYNTHASE*; *GPPS*, *GERANYL PYROPHOSPHATE SYNTHASE*.

One of the unresolved questions related to terpenoid metabolism is whether light differentially regulates the expression of genes involved in terpenoid biosynthesis (Dolzhenko et al., 2010). To have a better insight into the terpenoid biosynthesis pathway by UV-B light exposure, we exposed tea shoots to UVB to four different exposure periods as described in methodology and evaluated some of the main genes involved in the terpenoid biosynthesis (Figure 5A). We compared the expression levels of the genes in diterpenoid, monoterpenoid, and sesquiterpenoid pathways in all the UV-B treatments. Most of genes in diterpenoid biosynthesis were up-regulated across all the UV-B treated tea shoots. The majority of genes were down-regulated in monoterpenoid biosynthesis in 2 and 8 h exposure while upregulation was observed at 0.5 h treatment. Four genes in monoterpenoid biosynthesis pathway annotated as TPS13-1, TPS13-2, (+)-neomentholdehydrogenase1-1 (MNR1-1), (+) neomentholdehydrogenase1-2 (MNR1-2), were up-regulated (Supplementary Table S5). In sesquiterpenoid biosynthesis, we observed upregulation of most genes at 2 h followed by 8 h treatment. Significant downregulation of genes was observed in 8 h treatment followed by 2 h treatment (Figure 5B). UV-B irradiation induced up-regulation in all the genes in the IPP and DAMPP biosynthesis pathways in almost all the treatments except *HGMR-2* and *PMK-1, DXS* and *DXR* (Figure 5C). To validate the reliability of gene expression analysis in this study, 20 genes with potential roles in terpenoid biosynthesis, were selected for real-time qPCR analysis. Our real-time qPCR data showed that these genes expression patterns, matched the gene expression patterns observed in RNA-Seq analysis (Figure 5B). Majority of *TPS* genes which are crucial in terpenoid synthesis were not identified in our transcriptomic data. We identified 21*TPS* genes from tea genome for qPCR analysis to evaluate the effect of UVB exposure at different duration. Data from qPCR on *TPS* genes showed significant enhancement at 0.5 h with *TPS* genes annotated as linalool synthase strikingly enhanced (Figure 5D).

### UV-B Induced Changes in Specialized Metabolite Abundance

**FIGURE 6:**
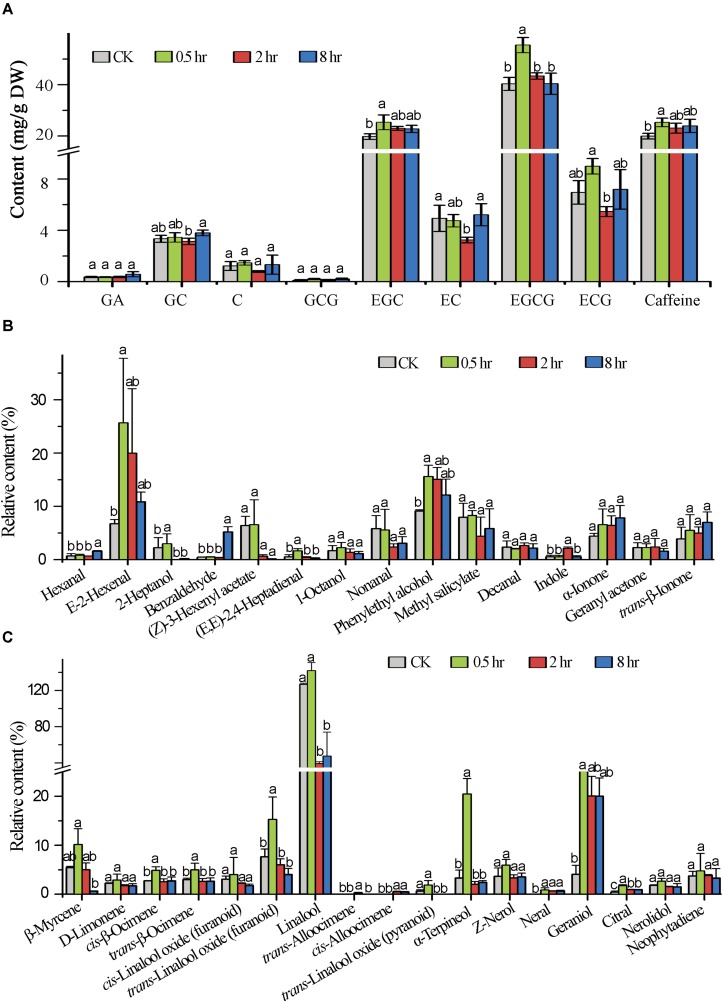
Induced metabolic changes in secondary metabolites biosynthesis pathway. **(A)** The concentration of major catechins in tea samples (0.5, 2, and 8 h) analyzed by HPLC. **(B,C)** Shows Volatile compound analysis GC-MS. Data shown are the average mean ± SE of three replicates (*n* = 3). Columns with different letters (a, b, ab) for the same compound had significant differences among the different treatments according to one way ANOVA and a fisher’s LSD test at *p* < 0.05.

In order to understand the impact of the UV-B treatment on flavor metabolite production in tea leaves, the abundances of flavor determinants catechins, caffeine, and terpenoid volatiles were quantified. The results showed that UV-B exposure for 0.5, 2, and 8 h resulted in differential accumulation of catechins. We analyzed catechins and majority of them including GA, ECG, EC, GCG and the most predominant EGCG in tea shoot tips flavor determinants had a statistically significant change at 0.5 h (*p* < 0.05) treatment as compared to 2 and 8 h treatment which showed reduced levels with an exception of GA, C, GCG and EGC which showed statistically significant increased levels in all the comparison groups (*p* < 0.05) (Figure 6A). All eight UV-B-upregulated genes (Supplementary Table S5) in the flavonoid pathway at 0.5 h treatment could be the reason why flavonoid levels were enhanced at the same period treatment.

In this study, we focused in determining the effect of different time durations of UV-B on terpenoid production. We analyzed the effect of different exposure periods of UVB on accumulation of terpenoids in tea shoots by quantifying different volatiles. We noted differential accumulation of terpenoids in the three treatments in comparison to control (0 h). A statistically significant accumulation of volatile compounds was noted at 0.5 h treatment. The majority of monoterpenoids were significantly decreased at 2 and 8 h treatments except geraniol which was significantly enhanced in all the three treatments. Linalool was significantly enhanced at 0.5 h treatment and decreased at 2 and 8 h treatments. We noted up-regulation of genes in monoterpenoid biosynthesis pathway at 0.5 h treatment (Supplementary Table S5), perhaps this could explain the increase in level of volatiles at 0.5 treatment. Other volatiles that were significantly enhanced in terpenoid pathway across the three comparisons were D-limonene, *cis*-linalool oxides, Z-nerol, and neral. The majority of volatiles in the non-terpenoid pathway were enhanced across the three comparison groups (Figures 6B,C) with significance enhancement noted at 0.5 h treatment. Our data clearly indicated that specialized metabolites are altered at short exposure (0.5 h) to UV-B.

## Discussion

### UV-B Induced Changes in UVR8-Mediated Signal Transduction Pathway

UV-B enhanced production of protectant screening phenolic compounds in plants have been well documented (Li et al., 1993; Rozema et al., 1997; Agati et al., 2011; Cañal et al., 2017), tea leaves contain much higher levels of polyphenols (16-30% of dry weight) than many other plants (Graham, 1992; Almajano et al., 2008). Some of these phenolics also function as flavor determinants for teas. UV-B radiation to tea plants may result in distinct responses from many other plant species and affect tea flavor. In this study, our transcriptomic analysis revealed that global transcriptomic alterations occurred in the tea shoots exposed to UV-B with the three different durations, similar to the previous findings (Casati et al., 2011). In addition, UVR8 was found down-regulated in all treatments in our experiments, as reported in maize (Casati et al., 2011) but contrary to the finding in *Arabidopsis* (Cloix et al., 2012; Heijde and Ulm, 2013; Vanhaelewyn et al., 2019). UV-B exposure altered the abundances of many specialized metabolites despite suppression of *UVR8* (Casati et al., 2011). Similar observatios were noted in our study where accumulation of flavonoid and volatile at 0.5 h treatment occurred. The stability of HY5 is enhanced by UVB irradiation leading to the monomerization of receptor *UVR8* which forms UVR8-COP1 complex accumulating in the nucleus enabling the transcription of genes derived from specific pathways responding to UV-B (Qian et al., 2016). *CHALCONE SYNTHASE (CHS), FLAVONOL SYNTHASE (FLS)* and numerous other genes belonging to diverse hormone and metabolic pathways are target genes for *HY5* which are up-regulated by UV-B (Gangappa and Botto, 2016), resulting in induction of flavonoid accumulation which acts as UV-B protectants (Demkura and Ballaré, 2012). In line with this, we observed the up-regulation of *HY5* at 2 and 8 h treatments in this study and the up-regulation of *FLAVONOL SYNTHASE (FLS)* at 8 h treatment. *CHALCONE SYNTHASE (CHS)* was down-regulated in our experiments contradicting early studies in other plants. The deferential induction of genes downstream of UVR8 could have been due to direct UV-B and not necessarily the suppressed *UVR8* and COP1 interaction as observed early by Jenkins (2009). We hypothesize the negative response of *UVR8* in tea could be part of feedback to suppress physiological changes stimulated by UV-B exposure due to already extraordinary accumulation of secondary metabolites in tea plants (Almajano et al., 2008; Punyasiri et al., 2004). Further studies need to be carried out to ascertain this hypothesis.

Through the regulatory module of COP1 and UVR8 (Rizzini et al., 2011; Jenkins, 2017), some transcriptional factors such as R2R3-MYB, bHLH, and WD40 (MBW ternary complexes) might be regulated, consequently controlling numerous enzymatic steps involved in the biosynthesis of flavonoid in plants (Mano et al., 2007; Zhao et al., 2013). Previous studies in tea plants have demonstrated the positive and negative regulatory role of R2R3-MYB in regulation of enzymes involved in flavonoid production (Zhao et al., 2013; Xu et al., 2015). *HY5* regulates the expression of *MYB12* as reported earlier which is a specific transcriptional factor for *FLAVONOL SYNTHASE (FLS)* responsible for accumulation of flavonoid under UV-B light exposure (Stracke et al., 2010). Transcription factor *MYB4* has been reported to be induced by UV-light which suppresses *C4H, 4CL, LAR, CHS* and *ANR2* in *A. thaliana, Brassica rapa*, and *C. sinensis* to mediate UV-B dependent anthocyanin and phenylpropanoid synthesis (Schenke et al., 2011; Li et al., 2017). In this study we observed up regulation of *MYB12* at 8 h corresponding to enhancement of *FLAVONOL SYNTHASE (FLS)* and divergent expression of *MYB4* corresponding to divergent alteration of genes in flavonoid synthesis pathway. *bHLH* is another family transcription factor in plants that plays several functions ranging from regulation of floral organ development to flavonoids biosynthesis (Sorensen et al., 2003; Ohno et al., 2011). Recently, transcriptional regulator *CsbHLH* was found to negatively regulates EGCG3”Me biosynthesis which is a major source of *O*-methylated catechin in tea. In this study, *bHLH* 62 was up-regulated at 8 h treatment. In addition, tea *MYB4a* negatively regulates *C4H*, *4CL*, *CHS*, *LAR*, and *ANR2* by physical interaction with target gene promoters (Li et al., 2017). In tobacco transgenics overexpressing tea *MYB4a*, a total of twenty (20) genes related to phenylpropanoid pathways are suppressed (Li et al., 2017), including the majority of UV-B suppressed genes (*CHS-1, CHS-2, CHS-3, DFR-1, DFR-2-DFR-3, ANR-1, F3′5′H-1, F3′5′H-2*, and *F3′H*) found in this study and likely to have been affected by *MYB4-2 and MYB4-4* which were upregulated. *MYB4-1* and *MYB4-3* was suppressed subsequently leading to enhancement of *4CL2-1, 4CL2-2, 4CL2-3, 4CL2-4, DFR-4, ANR-2, CHI, FSLI, ANS, LAR-1, LAR-2*, and *LAR-3,CCOAOMT-1, CCOAOMT-2, HST-1, HST-2, ANR-2, DFR-4, LAR-1* and *LAR-3.* The divergent expression of *MYB4* in this study could be the reason for transcriptional alterations on metabolic and structural genes in tea under UV-B exposure. Taken together these results validate the previous studies on how UV-B affects genes in UVR8-mediated signal transduction pathway altering secondary metabolites biosynthesis. Such transcriptional alterations might collectively be responsible for the UV-B induced metabolic and structural gene transcriptional alterations in tea plants.

### UV-B Induced Changes in Flavonoid Biosynthesis Pathway

Since a signaling metabolite must increase quickly in UVB irradiated shoots to induce transcriptome changes in response to stress within a short period (Mackerness, 2000), we predicted high level of specialized metabolites accumulation in treated shoots in 0.5 treatment. Our chemical analysis revealed that the majority of tea catechin including GA, ECG, EC, GCG and the most predominant EGCG in tea shoot tips as flavor determinants were enhanced at 0.5 h treatment compared to 2 and 8 h treatments, which significantly decreased with an exception of C, GCG, and EGC which was significantly enhanced in all the four treatments. There was no significant change at 2 and 8 h treatment compared to control in most of the tested metabolites, suggesting that the initial exposure to UVB led to different display in gene expression in tea shoots. This observation are in line with those made by Hectors et al. (2007) and Zhu et al. (2019). Even though the mechanisms underlying such differential abundance alterations in the tested catechins need further investigation, this finding is largely consistent with a previous report that a low fluence range of UV-B radiation results in decrease in all tested catechins throughout all time intervals (0, 30, 60, and 360 min), except for a slight increase in total catechin abundance detected at the interval of 30 min only in the tea cultivars “Fudingdabai” and “Yulan” (Zheng et al., 2008). Interestingly, the UV-B induced changes in the tested catechins in both cultivars are related to their original catechin abundances: the higher original catechin abundances in tea leaves, the less increase of UV-B induced catechin production after the first 30 min treatment and the more significant decrease of the UV-B induced catechin production after 360 min treatment. Besides, a recently published study indicated that long periods (1 to 16 days, 8 h per day) of a mild UV-B treatment (20 μW/cm^2^) applied to tea plants did not affect the abundance of the majority of flavonoid compounds, except for that of EGCG, kaempferol-7-*O*-glucoside, and GC (Liu L. et al., 2018). However, the detected GC enhancement only after 16 days of UV-B treatment relative to the control plants grown in hydroponic culture might result from a substantial decrease in all tested metabolites in control, not from UV-B treatment. In addition, UV-B irradiation reduces the abundance of anthocyanins in the berries of the red grape cultivar “Cabernet Sauvignon,” which generally contain high levels of polyphenolics (Sun et al., 2009). Whereas UV-B irradiation enhances polyphenol production in the berries of “Sauvignon blanc” (Liu et al., 2015), a white grape cultivar usually containing low levels of polyphenols. Taken together, these results suggest that the distinct responses of tea plants after UV-B radiation might result from their constitutively high levels of polyphenols. Such alterations in the flavor determinant metabolites such as catechins might affect tea beverage astringency, bitterness (Chaturvedula and Prakash, 2011; Feng et al., 2014) and biological effects on consumers. Further investigation on the mechanism underlying the difference in UV-B induced metabolite production between tea and some other plants is required.

### UV-B Induced Changes in Volatile Terpenoid Pathway

In plants, volatile terpenoids can be synthesized through the mevalonate and methylerythritol phosphate (MEP) pathways (Schwab et al., 2008). Genes in the MVA pathway such as *AACT1* have been reported to be triggered by abiotic stresses in alfalfa (Soto et al., 2011). Studies in *populus trichocarpa* have indicated that overexpression *PtHMGR* resulted in the increase in content of terpenoids including lycopene, gibberellic acid (GA), ABA, and carotenes indicating its role in terpenoid biosynthesis regulation (Wei et al., 2019). *AACT1 HMGR*, *HMGS*, and *DXS*, *PMK* and *MVK* from either the MVA or MEP pathway are all enhanced by light, jasmonic acid and ethylene (Hemmerlin et al., 2012). All these genes except *HGMR-2*, *PMK-1 DXR and DXS* were found to be enhanced by UV-B treatment in our work. Our qPCR data showed significant enhancement at 0.5 h with *TPS* genes annotated as linalool synthase strikingly enhanced. This could explain the accumulation of linalool at 0.5 h compared to other treatments. Four genes in monoterpenoid biosynthesis pathway annotated as MNR1-1, MNR1-2, TPS13-1, and TPS13-2 were up-regulated at 0.5 h treatment consistent with the UV-B enhanced monoterpenoids. Our data suggested that UV-B differentially affected volatile terpenoid biosynthesis with great potential for tea aroma improvement.

Terpenoids play a key role in the survival of plants as well as beneficial to human health. We analyzed the effect of different exposure periods (0, 0.5, 2, and 8 h) of UVB on accumulation of terpenoids in tea shoots. UV-B induced changes in metabolic and transcriptional profiles of terpenoid volatiles in tea shoot-tips. We examined whether a different time duration of UV-B irradiation alone can affect the expression of *TPS* genes and the production of related terpenoid volatiles in the intact tea leaves, rather than the excised leaves, in which enhancement of numerous volatiles (including linalool and nerolidol) and the expression of the genes, respectively, encoding ß-glucosidase and ß-primeverosidase were reported after UV-B exposure for 2 h (Jang et al., 2010). Our metabolic profiling showed that UV-B could induce the production of several potent monoterpenoid odorants such as linalool and geraniol in the intact tea leaves. We observed differential accumulation of terpenoids in the three treatments compared to control (0 hr). A significant accumulation of volatile compounds was observed at 0.5 h treatment. The majority of monoterpenoids were significantly decreased at 2 and 8 h treatment except geraniol which was significantly enhanced in all the treatments. Linalool was significantly enhanced in 0.5 h treatment and decreased at 2 and 8 h treatment. This is consistent with transcriptomic changes in monoterpenoid biosynthesis pathway in which most of the genes were suppressed at 2 and 8 h treatment and enhanced at 0.5 h treatment. This result is also in agreement with the two previously published reports that multiple volatile terpenoids can be enhanced in grapes and peach due to UV-B treatment (Gil et al., 2012; Liu et al., 2017). Our data suggest that a short period of UV-B exposure for 0.5 h could potentially improve tea aroma. Taken together, these findings suggest that alteration in the expression of genes responsible for terpenoid biosynthesis contributes to metabolic regulation of tea under UV-B irradiation.

## Conclusion

We have found that UV-B induced distinct transcriptomic alterations in the UVR8-signal transduction, phenylpropanoids and volatile terpenoids pathways in *Camellia sinensis.* Numerous studies on effect of high fluence UV-B on plants including Arabidopsis have indicated that UVR8 is up-regulated influencing signal transduction downstream of UV-B perception through to the induction of UV-B protectants such as flavonoids. This is in contrast with our observation in this study where UVR8 was down regulated in all the treatments. There was no significant increase in specialized metabolites except at 0.5 h where major flavonoids and terpenoids were enhanced. However, despite enhanced flavonoid and terpenoid accumulation at 0.5 hr, we observed divergent expression of genes in flavonoid biosynthesis pathway including transcription factor *MYB4*. Tea is extra ordinarily rich in phenolic compounds, it will be interesting to understand molecular mechanism underlying down regulation of *UVR8* in tea under UV-B exposure without significantly influencing accumulation of flavonoids as observed at 2 and 8 h treatment. The accumulation of special metabolites at shorter period of treatment indicates that metabolite signals are induced in response to stress as a defense mechanism. Stability of metabolites after 2 h duration suggested that the plant had acclimated to stress and displayed different expression profiles to those exposed over shorter duration. The 0.5 h treatment showed accumulation of key flavor determinants in tea which can be practically applied at industrial level in tea industry.

## Data Availability Statement

The datasets generated for this study can be found in NCBI SRA data IDs: SRR10768500–SRR10768511 and BioProject ID PRJNA597433.

## Author Contributions

LS and H-CZ performed the experiments, conducted the data analysis, and manuscript draft preparation. Z-XH started the project and prepared the samples for RNA-Seq. SW conceived and designed the study, analyzed the data, and finalized the manuscript.

## Conflict of Interest

The authors declare that the research was conducted in the absence of any commercial or financial relationships that could be construed as a potential conflict of interest.

## Supplementary Material

FIGURE S1 Shows the effect of different time period exposure of UV-B light on stress inducement.

FIGURE S2 Gene structure and phylogenetic tree of *MYB4* variants in UVB treated tea shoots.

FIGURE S3 Statistics of Differential expressed genes among different treated samples.

TABLE S1 Summary of RNA-seq statistical Assembly.

TABLE S2 Primers used in this study for qPCR validation of RNA-seq.

TABLE S3 Top Total Significant KEGG pathways associated with dififferentially expressed genes (DEGs) induced by different treatments.

TABLE S4 Selected stress UV-B induced genes in tea shoot tips.

TABLE S5 Top Up and Downregulated KEGG classifications associated with dififferentially expressed genes (DEGs) induced by different treatments.
