# Supplementary figures and images for: UV-B Induces Distinct Transcriptional Re-programing in UVR8-Signal Transduction, Flavonoid, and Terpenoids Pathways in Camellia sinensis
Source: Front Plant Sci. 2020 Mar 3;11:234. doi: 10.3389/fpls.2020.00234 (PMC7062797; doi:10.3389/fpls.2020.00234)

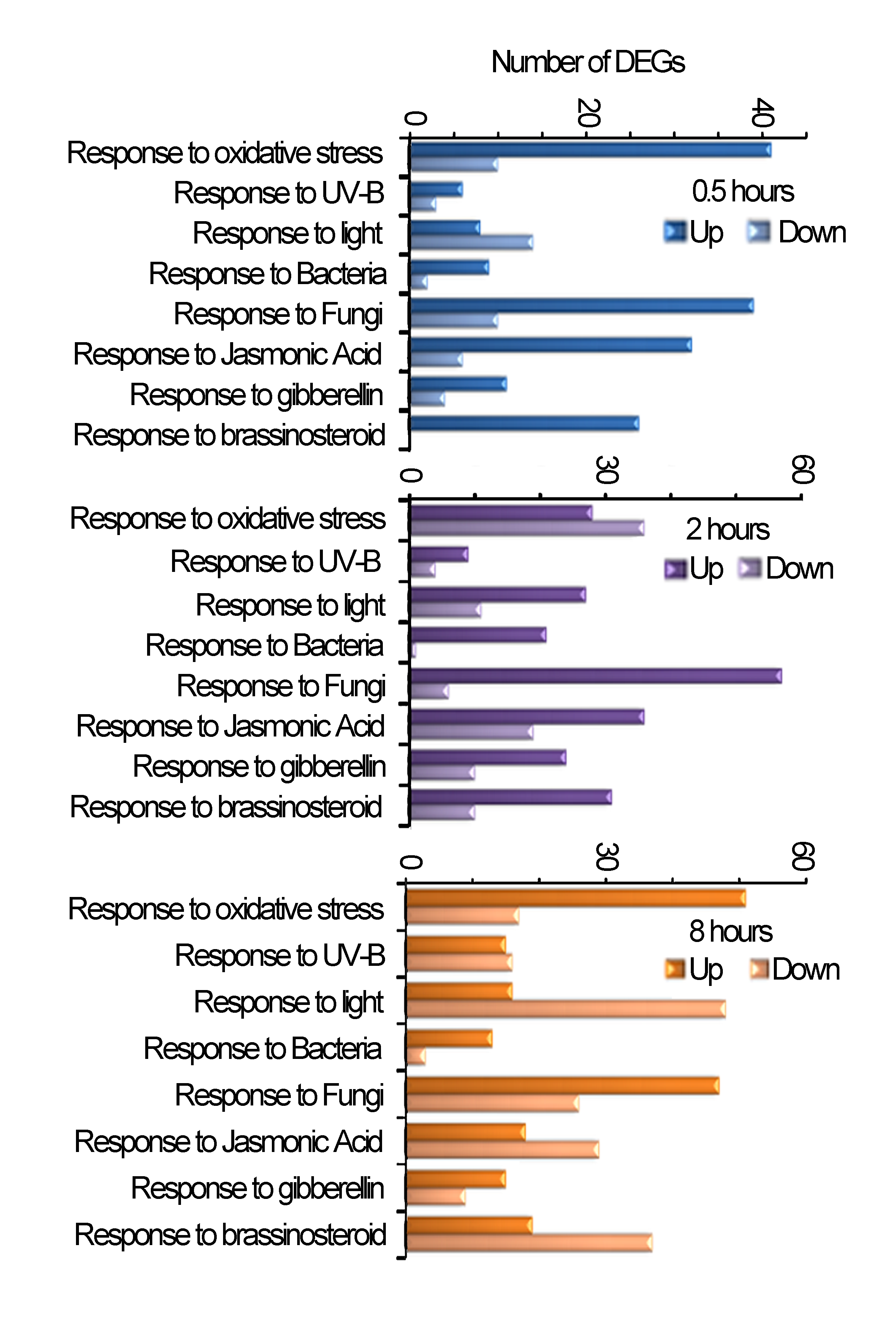

Supplement: FIGURE S1 — Shows the effect of different time period exposure of UV-B light on stress inducement. [file Image_1.TIF]

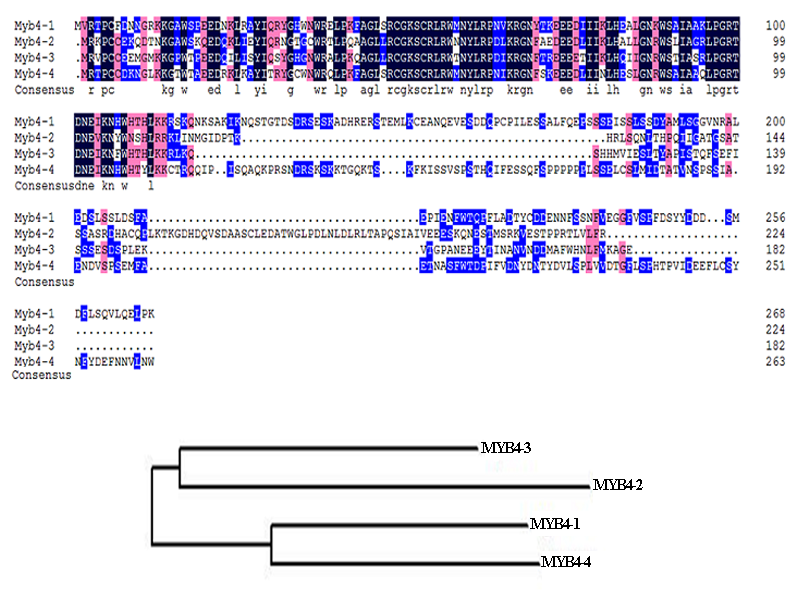

Supplement: FIGURE S2 — Gene structure and phylogenetic tree of MYB4 variants in UVB treated tea shoots. [file Image_2.TIF]

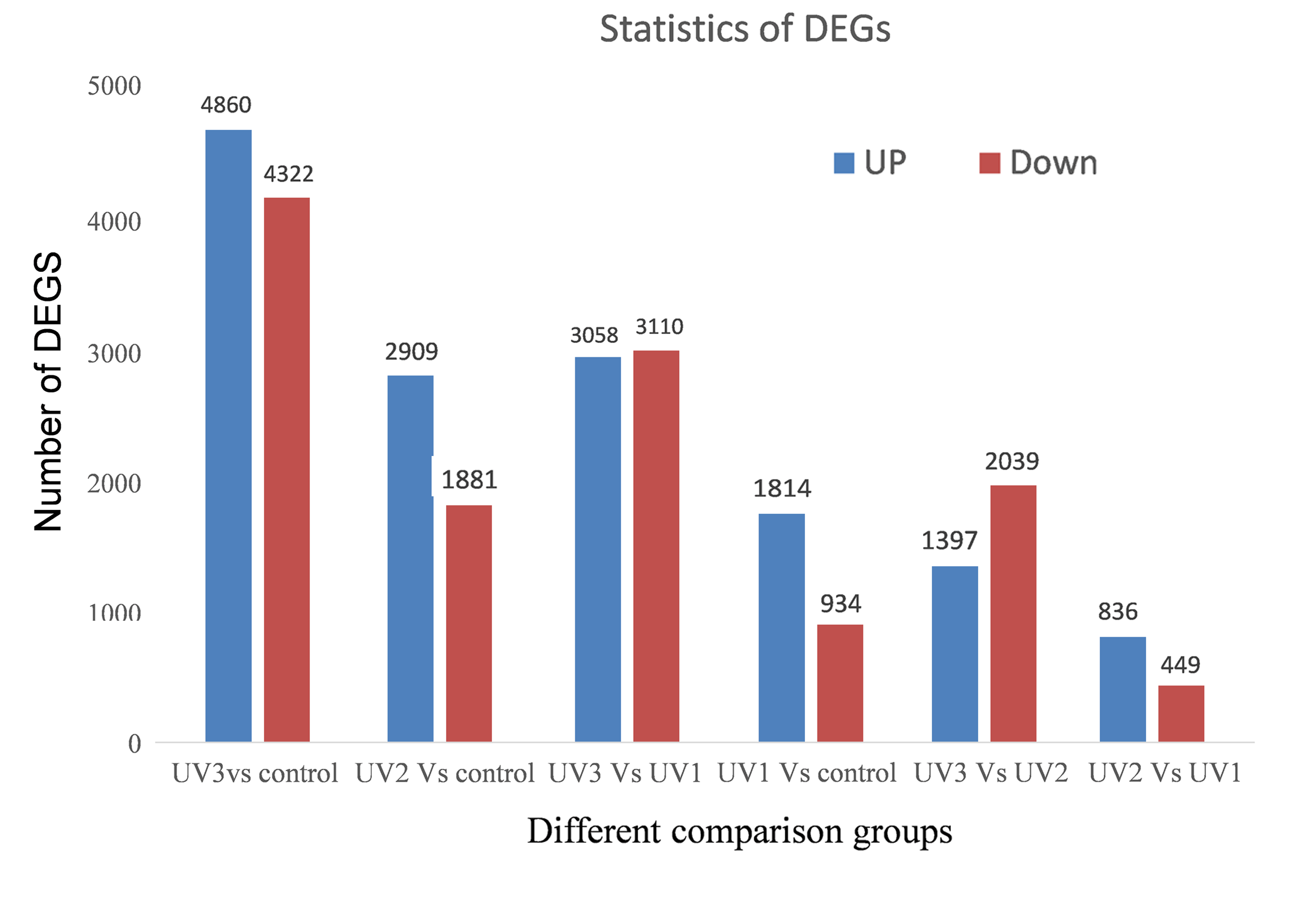

Supplement: FIGURE S3 — Statistics of Differential expressed genes among different treated samples. [file Image_3.TIF]
